# Advancing Symbiodiniaceae Functional Ecology Through a Trait‐Based Framework

**DOI:** 10.1002/ece3.74237

**Published:** 2026-08-31

**Authors:** Luna Mayura F. Bauer, Michelle Amario, Luiza P. Campos, Aline C. Shimada, Marina T. Botana, Sarah W. Davies, Amana G. Garrido, Arthur Z. Güth, Guilherme O. Longo, André L. Luza, Matthew R. Nitschke, John E. Parkinson, Flávia M. P. Saldanha‐Corrêa, Christian R. Voolstra, Carla Zilberberg, Miguel Mies

**Affiliations:** ^1^ Instituto Oceanográfico Universidade de São Paulo São Paulo Brazil; ^2^ Instituto de Biociências Universidade de São Paulo São Paulo Brazil; ^3^ Institute of Environmental Medicine Karolinska Institutet Stockholm Sweden; ^4^ Department of Biology Boston University Boston Massachusetts USA; ^5^ Departamento de Invertebrados Museu Nacional, Universidade Federal do Rio de Janeiro Rio de Janeiro Brazil; ^6^ Instituto Coral Vivo Santa Cruz Cabrália Brazil; ^7^ Departamento de Oceanografia e Limnologia Universidade Federal do Rio Grande do Norte Natal Brazil; ^8^ Department of Biology University of Massachusetts Boston Boston Massachusetts USA; ^9^ UMR Biodiversité Gènes et Communautés INRAE, Université de Bordeaux Pessac France; ^10^ Australian Institute of Marine Science Cape Cleveland Queensland Australia; ^11^ Department of Integrative Biology University of South Florida Tampa Florida USA; ^12^ Department of Biology University of Konstanz Konstanz Germany; ^13^ Instituto de Biodiversidade e Sustentabilidade, Universidade Federal do Rio de Janeiro Macaé Brazil

**Keywords:** attribute, climate change, coral reefs, functional diversity, *Symbiodinium*, symbiosis

## Abstract

Symbiodiniacean dinoflagellates are fundamental to the functioning of coral reefs, underpinning primary production, nutrient cycling, and calcification through intimate intracellular symbioses with corals and other marine invertebrates. The identity and functional traits of these endosymbionts strongly influence host physiology, particularly thermal tolerance and stress resilience. Despite their ecological importance, Symbiodiniaceae have not yet been characterized within a formal functional ecology framework. Trait‐based functional ecology enables standardized comparative analysis using metrics including functional diversity (richness, evenness, and divergence) and redundancy, which are critical for assessing ecosystem stability and vulnerability. Progress on this front requires elucidating clearly defined traits for Symbiodiniaceae. Here, we propose a standardized functional trait framework for these organisms. We identify key conceptual and methodological barriers that have hindered the integration of Symbiodiniaceae into formal descriptions of functional ecology, including unresolved species boundaries, limited trait standardization, and the context‐dependent expression of traits in hospite versus in vitro. Building on principles from trait‐based ecology, supported by empirical data and experimental measurements, we define and propose a set of 19 functional traits categorized into nine core functions: photosynthesis, photoprotection, cellular growth, population growth, energy reserves and composition, symbiotic relationship, nitrogen assimilation, ecological plasticity, and thermal tolerance. These traits capture fundamental dimensions of algal symbiont performance, including resource acquisition, stress tolerance, metabolic allocation, and host interaction, providing a foundation for calculating functional diversity metrics. Integrating Symbiodiniaceae into a functional trait framework will improve our capacity to assess functional redundancy, vulnerability, and resilience of coral reefs, ultimately strengthening forecasts of reef persistence under ongoing climate change.

## Graphical abstract

Symbiodiniaceae dinoflagellates are central to coral reef functioning, supporting primary production, nutrient cycling, calcification, and influencing coral thermal tolerance through intracellular symbiosis. Despite their ecological importance, these symbionts have not yet been incorporated into a formal functional ecology framework due to challenges such as unresolved species boundaries, limited trait standardization, and context‐dependent trait expression. Here, we propose a standardized trait‐based framework defining 19 functional traits across eight core functions to enable functional diversity analyses and improve assessments of coral reef resilience under climate change.

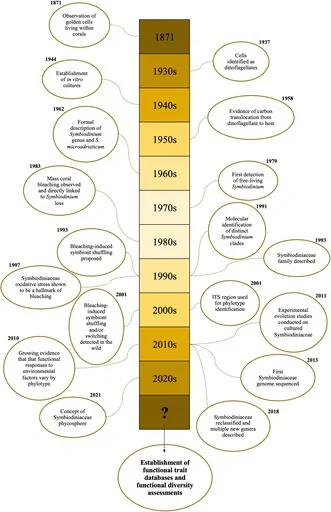

## 1 Introduction: The Ecological Importance of Symbiodiniaceae

**FIGURE 1:**
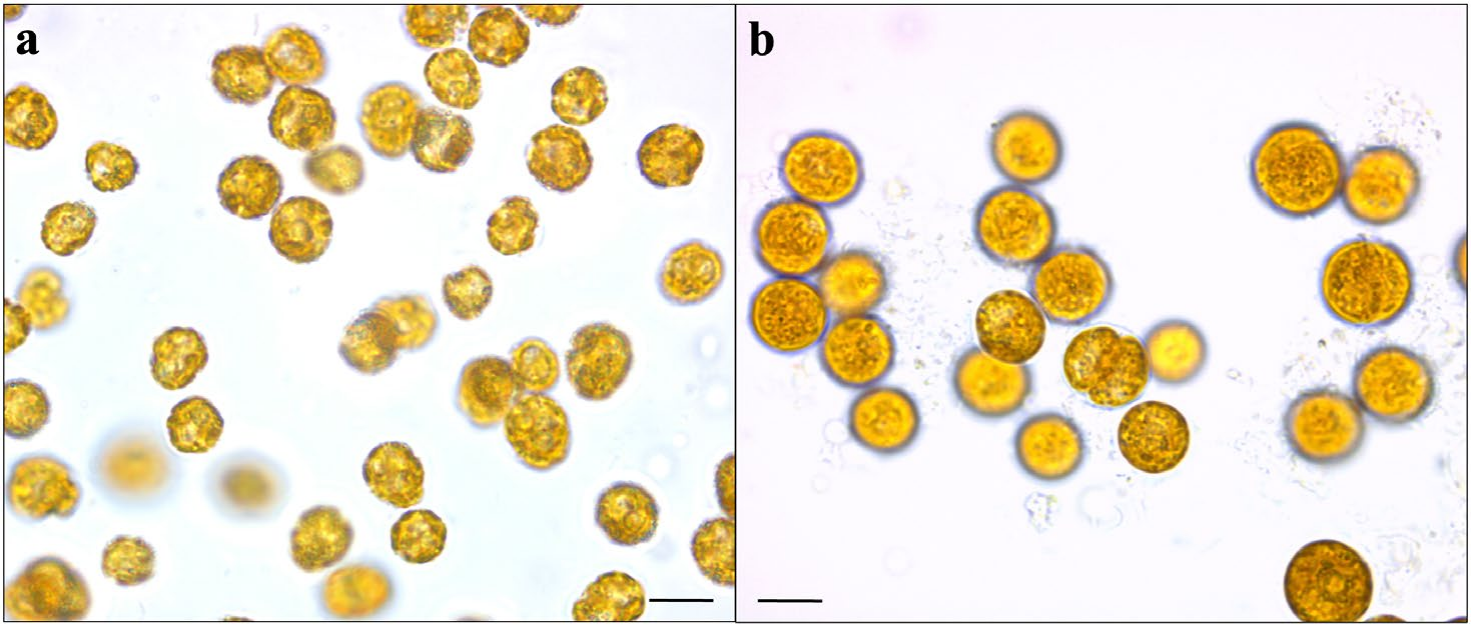
Symbiodiniaceae cells cultured at the Oceanographic Institute of the University of São Paulo, under a microscope (scale bar = 8 μm). (a) *Cladocopium goreaui*, ITS2 type profile C1, (b) *Fugacium kawagutii*, ITS2 type profile F1.

Photosynthetic dinoflagellates of the family Symbiodiniaceae (Figure 1) are critical structural elements of coral reefs (LaJeunesse et al. 2018). Through highly specific intracellular symbioses with cnidarians, particularly reef‐building corals, these symbionts form the basis of coral reef ecosystems. This association is the foundation of the coral holobiont (the conglomerate of the animal host and its diverse assemblage of associated microbes), enabling these organisms to thrive in oligotrophic tropical waters and, thereby, to build a diverse and complex ecosystem (Crossland et al. 1991; Davy et al. 2012; Vollmer et al. 2013; Cardini et al. 2015; Adams et al. 2020). Symbiodiniaceae provide photosynthetically fixed carbon to cover almost entirely the metabolic requirements of their host (Muscatine et al. 1981; Tremblay et al. 2012; Tanaka et al. 2018). Besides, Symbiodiniaceae recycle nitrogen and phosphorus through the assimilation of coral metabolic waste and uptake of nitrogen from the seawater (Rädecker et al. 2015; Ferrier‐Pagès et al. 2016; Li et al. 2023). In addition, Symbiodiniaceae photosynthetic activity enhances coral calcification by providing energy, elevating oxygen concentrations, and increasing pH within the host, thus promoting the precipitation of aragonite that forms the coral skeleton and, consequently, the reef framework (Gattuso et al. 1999; Cohen et al. 2016; Galli and Solidoro 2018; Venn et al. 2025). Therefore, these symbionts modulate key processes that underpin the structure and function of coral reefs (Baker 2003; Stat et al. 2006; Fabina et al. 2013; Roth 2014).

The association between corals and Symbiodiniaceae has been the subject of much investigation, particularly in the context of climate change. As large‐scale coral bleaching and mortality has increased in prevalence, so has a reduction in global marine biodiversity and ecosystem services, such as fisheries, tourism, and coastal protection (Woodhead et al. 2019; Eddy et al. 2021). The increasing intensity, duration, and frequency of marine heatwaves (Oliver et al. 2018; Skirving et al. 2019; Hoegh‐Guldberg et al. 2023; Destri et al. 2025; Eakin et al. 2026) have led to widespread coral bleaching, the physical whitening that signifies the loss of algal symbionts, and their pigments resulting from the breakdown of the coral–Symbiodiniaceae association (Glynn 1996; Hughes et al. 2018). This dysbiosis creates an energetic deficit for the host that may, eventually, result in coral death (Grottoli et al. 2006; Rädecker et al. 2021, 2023). A major determinant of the overall thermal tolerance of the coral holobiont is the algal symbiont assemblage, given the pronounced differences in thermal tolerance among Symbiodiniaceae (Baker et al. 2004; Berkelmans and Van Oppen 2006; Sampayo et al. 2008; Suggett et al. 2017; Swain et al. 2017; Hoadley et al. 2021; Van Nynatten et al. 2025). Therefore, the diverse physiological response spectra of Symbiodiniaceae with respect to changing physico‐chemical conditions have critically shaped the persistence of coral reefs in the past and will continue to do so in the future. However, the link between taxonomy and functional responses of Symbiodiniaceae remains poorly resolved, limiting the ability to formally link species composition to reef ecosystem functioning (Davies et al. 2023).

## 2 The Current Knowledge of Symbiodiniaceae Taxonomic and Functional Diversity

**FIGURE 2:**
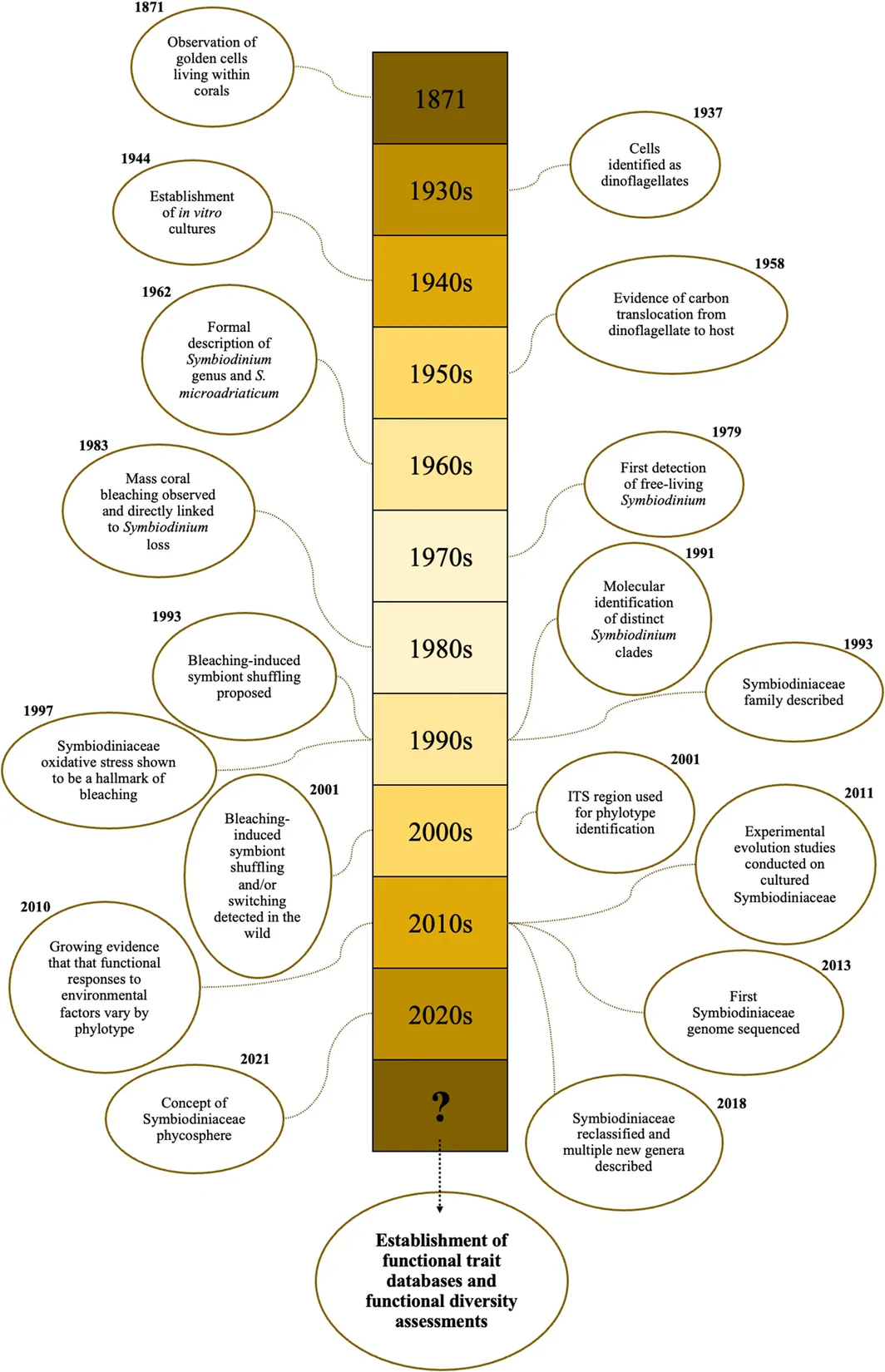
Timeline with milestones illustrating the progression of scientific knowledge on Symbiodiniaceae, from the initial description to recent conceptual and molecular advances. The production of functional trait databases and functional diversity models is still lacking. Information taken from Cienkowski (1871), Hovasse (1937), Kawaguti (1944), Muscatine and Hand (1958), Freudenthal (1962), Loeblich and Sherley (1979), Glynn (1983), Rowan and Powers (1991), Fensome et al. (1993), Buddemeier and Fautin (1993), Lesser (1997), Baker (2001), LaJeunesse (2001), Finney et al. (2010), Huertas et al. (2011), Shoguchi et al. (2013), LaJeunesse et al. (2018), and Garrido et al. (2021).

The understanding of Symbiodiniaceae diversity has undergone a significant shift over the last few decades (Figure 2). Initially, Symbiodiniaceae were assumed to comprise a single panmictic species, *Symbiodinium microadriaticum* (Freudenthal 1962). However, the application of molecular tools has fundamentally transformed this perspective. Using genetic markers, it was found that the original genus “*Symbiodinium*” *sensu lato* harbored tremendous genetic diversity (comparable to taxonomic orders in other dinoflagellate groups) and was initially divided into major “clades,” labeled A to J, to account for such divergence (Rowan and Powers 1991a, 1991b; Pochon et al. 2014; Yorifuji et al. 2021). In 2018, these “clades,” which had always been intended as a temporary construct, were formally recognized as broadly equivalent to genera, and the family Symbiodiniaceae was erected (LaJeunesse et al. 2018). To date, 11 genera have been formally described: *Breviolum*, *Cladocopium*, *Durusdinium*, *Effrenium*, *Freudenthalidium*, *Fugacium*, *Gerakladium*, *Halluxium*, *Miliolidium*, *Philozoon*, and *Symbiodinium sensu stricto* (LaJeunesse et al. 2018, 2022; Nitschke et al. 2020; Pochon and LaJeunesse 2021). Although only ~40 Symbiodiniaceae species have been formally described, thus far, hundreds of species are assumed to exist, with large population sizes and regional genetic diversity (Parkinson et al. 2025). Symbiodiniaceae diversity is most commonly assessed using the Internal Transcribed Spacer 2 (ITS2) rDNA region, a multicopy noncoding molecular marker, often in the form of majority ITS2 sequence variants (LaJeunesse 2001; Sampayo et al. 2009) or integrated ITS2 type profiles (via the SymPortal pipeline; Hume et al. 2019). However, these classifications do not correspond directly to species boundaries, which still require multilocus genotyping for formal taxonomic description (Davies et al. 2023). Rather, multiple ITS2 type profiles may correspond to a single species. In fact, the current understanding is that ITS2 type profiles resolve subspecies lineages or even genotypes (Hume et al. 2019; Dubé et al. 2023). In this context, the absence of universal genetic divergence cutoffs to resolve species boundaries within the Symbiodiniaceae complicates comparisons across studies. Nevertheless, the standardized use of ITS2 type profiles, through the SymPortal pipeline, has facilitated comparability across datasets and studies (Fiesinger et al. 2025). Current efforts now seek to explicitly resolve how ITS2 type profiles map onto Symbiodiniaceae lineages and genotypes. Conversely, progress in resolving taxonomic relationships has not been accompanied widely by efforts to define Symbiodiniaceae traits to their ascribed taxonomic diversity. Even in instances where defined species or algal cultures are available (e.g., Röser et al. 2024), a general paucity of physiological trait data is apparent. In cases where functional traits are investigated, high variability is observed within defined species (Parkinson and Baums 2014; Hawkins et al. 2016; Díaz‐Almeyda et al. 2017).

Another obstacle is the lack of functional diversity assessments for Symbiodiniaceae, largely resulting from the absence of standardized and clearly defined functional traits. Once multiple functional traits become available, they can be incorporated into functional frameworks to derive several functional metrics. These formal functional models use the distribution of species in trait space (effectively the map of ecological niches—Lamanna et al. 2014; Carmona et al. 2024) to calculate specific indices that represent different facets of functional diversity (e.g., functional richness, evenness, divergence, and originality). These functional indices can be related to environmental and geographical variables through statistical models or contrasted with null expectations in simulations to assess ecosystem stability and vulnerability to human‐mediated and ecological drivers of change (Mouillot et al. 2014; Waechter et al. 2022). High functional diversity is often linked to greater ecosystem resilience, as it promotes functional complementarity and enhances the capacity of a given assemblage to maintain ecological functions under environmental disturbances (Mouillot et al. 2014; Nash et al. 2016). For instance, coral assemblages composed of species with diverse growth forms and light‐harvesting morphologies, as well as fish assemblages encompassing a wide array of herbivores, detritivores, and predators, collectively contribute to essential processes such as nutrient cycling, bioerosion, and algal overgrowth control, which enhance ecosystem resilience (Bellwood et al. 2004; Darling et al. 2012; Richardson et al. 2018). Building on the concept of functional diversity, functional redundancy highlights how multiple species within a community can perform similar ecological roles (Rosenfeld 2002; Loreau 2004). This redundancy contributes to both ecological resistance and resilience by ensuring that key functions persist even when particular species decline or go extinct (Elmqvist et al. 2003; Nyström 2006). When several species share overlapping functional traits, they can compensate for one another, reducing the risk of functional loss under environmental disturbance causing species losses. This buffering effect is clearly illustrated in Caribbean coral reefs, where parrotfishes and sea urchins both fulfill the role of herbivores, removing algae and maintaining open substrate, essential processes for coral recruitment and reef accretion (Mumby et al. 2007; Burkepile and Hay 2008; Bonaldo et al. 2014). In such systems, high redundancy minimizes the likelihood of functional collapse following species loss, thereby helping prevent shifts to degraded ecological states (Walker 1992; Hoey and Bellwood 2009). However, evaluating the role of functional redundancy in Symbiodiniaceae is currently constrained by the limited availability of data on the functional traits of individual taxa. For instance, it remains unclear whether the strength of the coral–algal mutualism and host performance are preserved when locally extinct symbionts are replaced by more generalist taxa.

## 3 The Case for Symbiodiniaceae Trait Databases

Trait databases represent the compilation and standardization of individual‐ and species‐level measurements of traits (i.e., attributes) relevant to organismal performance and function (Violle et al. 2007). These databases cover many species or lineages (Leiva et al. 2025), and functional ecology studies can be conducted across taxa and ecosystems (Pinsky et al. 2022; Luza et al. 2023). By querying these databases, “functional framework” or “trait‐based” approaches can be implemented (e.g., Petchey and Gaston 2006; Villéger et al. 2008; Mouillot et al. 2013; Green et al. 2022) to determine community‐level descriptors of functional diversity, such as functional richness, evenness, redundancy, and turnover (Mason et al. 2005; Halpern and Floeter 2008; Rodriguez et al. 2016). Such trait‐based approaches have proven effective for linking species traits to ecosystem function and resilience, thus enabling predictions of how shifts in community composition may influence key processes (Douglas et al. 2017; Gladstone‐Gallagher et al. 2019; Martini et al. 2021; Carturan et al. 2022; Green et al. 2022).

Trait databases and trait‐based functional models have become central tools in modern ecology for understanding how organisms interact with their environment and how communities respond to environmental change. Functional traits are morphological, physiological, biochemical, or behavioral characteristics that define how organisms acquire and utilize resources, withstand environmental stressors, grow, reproduce, and ultimately affect ecosystem functioning (McGill et al. 2006; Violle et al. 2007; Díaz et al. 2016; Kearney et al. 2021; Alfaro‐Lucas et al. 2024). Specifically, functional traits describe how organisms respond to environmental conditions—such as variation in photosynthetic efficiency along light gradients—and how they influence their environment and interact with other organisms, for example, through host specificity in symbiotic relationships, ultimately affecting host productivity (McGill et al. 2006; Cadotte et al. 2015; Dawson et al. 2021).

Functional trait databases have been developed recently for reef fish, corals, and macroalgae. These databases compile information on species functional traits and integrate data from multiple studies and geographic regions, enabling comprehensive global and regional data‐mining analyses. The “Traits of Fish” (TOFF) database (Lecocq et al. 2019), for example, is an online resource accessible via R scripts that encompasses dozens of fish functional traits, such as maximum length, age at maturity, and body shape. Additional fish functional trait datasets are available in the Supporting Information of published articles (e.g., Parravicini et al. 2021; Quimbayo et al. 2021). For corals, the “Coral Traits” database (Madin et al. 2016) compiles information on 172 traits for more than 5000 species, encompassing traits such as growth form, corallite width, depth range, and even associated Symbiodiniaceae genera (“clades”). Primary producers, such as macroalgae, also have dedicated databases, such as “Algae Traits,” which compiles data on traits including lifespan, dispersion mode, gametophyte arrangement, and cytomorphology for European seaweeds (Vranken et al. 2023). For Symbiodiniaceae, however, functional trait databases remain entirely absent.

## 4 Defining a Map of Meaningful Functional Traits for Symbiodiniaceae

Differences among Symbiodiniaceae species manifest in a range of physiological traits, including photosynthetic performance, thermal tolerance, oxidative stress response, and nutrient utilization, all of which influence the stability and resilience of the coral–algal symbiosis under a changing environment (Robison and Warner 2006; Suggett et al. 2008; Howells et al. 2012; Loures et al. 2026). As a result, the presence of a specific symbiont lineage within a host can confer an adaptive advantage, enhancing the capacity of the holobiont to withstand and recover from disturbances (Stat and Gates 2011; Cunning et al. 2015).

Members of the family Symbiodiniaceae exhibit a wide range of functional responses to environmental conditions, most notably with respect to thermal tolerance; such variation is both inter‐ and intra‐specific. For example, *Symbiodinium microadriaticum* withstands high temperatures by maintaining antioxidant defenses and photochemical stability under heat stress, whereas *S. tridacnidorum* is thermally sensitive and *S. necroappetens* is heat‐tolerant or ‐sensitive depending on the clonal strain under consideration (Díaz‐Almeyda et al. 2017; Swain et al. 2017; Mies et al. 2018, 2025). Additionally, some symbiodiniaceans are adapted to high irradiance in shallow waters (e.g., *C. infistulum*; Gonzalez‐Pech et al. 2024), while others are adapted to low‐light conditions found in turbid or mesophotic locations (e.g., multiple lineages within *Cladocopium* and the C3 radiation) (LaJeunesse 2005; Chan et al. 2009; Bongaerts et al. 2011; Rouzé et al. 2021). Therefore, the diversity of environmental conditions likely shaped the functional differentiation and ecological specialization observed among Symbiodiniaceae lineages.

Symbiodiniaceae also display distinct ecological strategies when interacting with hosts. Some are specialists and exhibit strong host specificity, such as *Cladocopium pacificum* (restricted to pocilloporid coral hosts; Turnham et al. 2021), *Breviolum dendrogyrum* (restricted to the Caribbean pillar coral *Dendrogyra cylindrus*; Lewis et al. 2019), and *Gerakladium endoclionum* (restricted to sponges in the family Clionaidae; Ramsby et al. 2017). Others, however, are generalists and form symbioses with multiple host species, sometimes across different phyla (e.g., *C. proliferum* and *C. vulgare* ; Butler et al. 2023). Moreover, some symbiodiniaceans are highly effective mutualistic partners, translocating substantial amounts of organic carbon to their hosts, whereas others contribute little (Loram et al. 2007; Stat et al. 2008; Leal et al. 2015). In fact, some are parasitic opportunists, such as *S. necroappetens*, which monopolizes diseased coral tissue (Lesser et al. 2013; LaJeunesse et al. 2015; Villela et al. 2025). Finally, some species, such as *Effrenium voratum*, do not form stable symbioses, and are functionally free‐living symbiodiniaceans. These examples illustrate just a fraction of the remarkable diversity in functional adaptations and responses within Symbiodiniaceae.

## 5 A Proposed Set of 19 Ecologically Relevant Symbiodiniaceae Functional Traits

**FIGURE 3:**
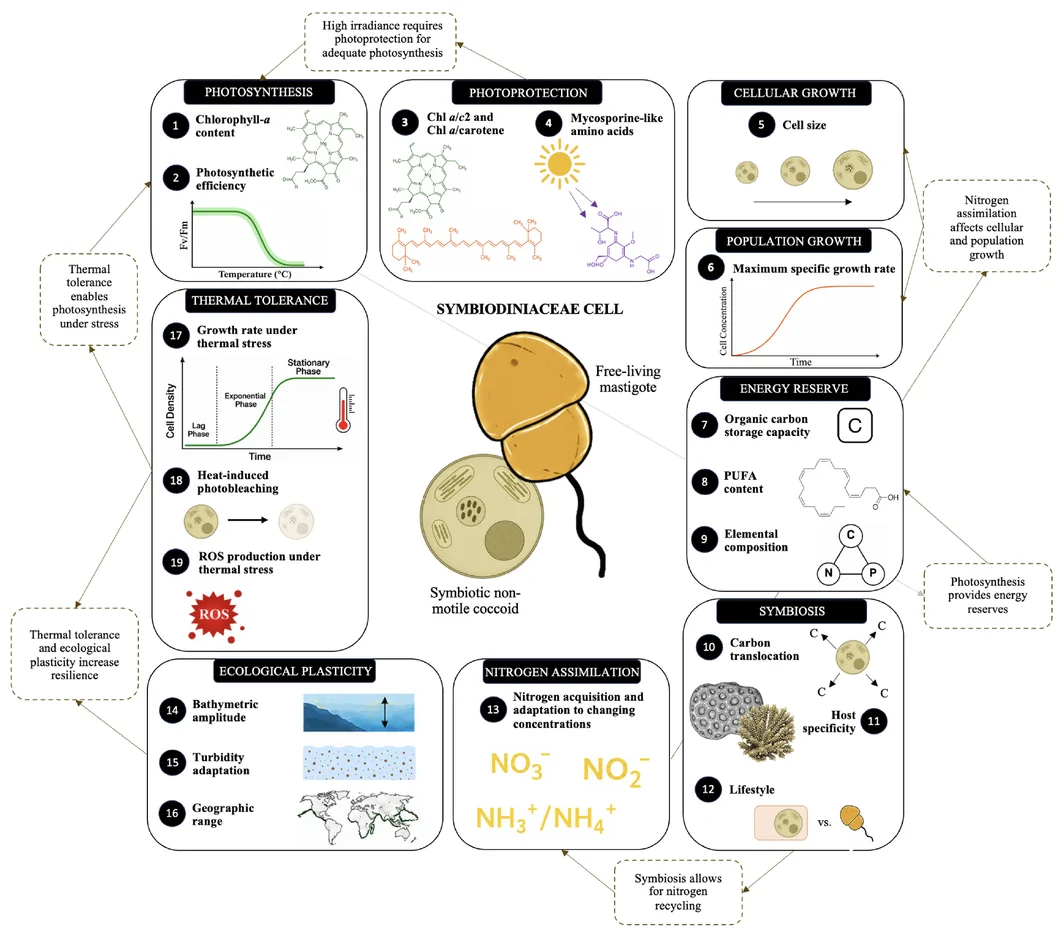
Conceptual diagram of the 19 functional traits proposed in this study for Symbiodiniaceae, grouped into nine core functions that together describe major dimensions of physiology, ecological performance, and symbiotic behavior: Photosynthesis, photoprotection, cellular growth, population growth, energy reserve and composition, symbiosis, nitrogen assimilation, ecological plasticity, and thermal tolerance. These functions are not independent but are mechanistically and ecologically interconnected (see dashed outside boxes), illustrating a network of feedbacks and dependencies among functions and reflecting the integrated nature of Symbiodiniaceae phenotypes across free‐living and symbiotic contexts.

A well‐defined functional trait should (i) capture a biologically relevant dimension of organismal performance, (ii) be quantifiable through standardized and replicable methods, and (iii) demonstrate a causal link to an ecological function such as nutrient exchange, stress tolerance, or symbiotic stability (McGill et al. 2006; Violle et al. 2007). Based on these principles, we propose a set of 19 functional traits (Table 1, Figure 3) encompassing eight broad functional categories that are ecologically meaningful and operationally measurable to support the establishment of functional trait databases and models for Symbiodiniaceae. Many of these traits draw on well‐established, routinely applied metrics in coral reef studies, while others remain comparatively underrepresented in the literature.

**TABLE 1:** Proposed set of 19 ecologically relevant and operationally measurable functional traits of Symbiodiniaceae, distributed across nine functional categories, for integration into a trait database aimed at supporting functional modeling within Symbiodiniaceae.

Trait	Function	Unit/Metric	Type	Experimental assessment	Relevance
Chlorophyll‐*a* content	Photosynthesis	pg chl‐a cell^−1^	Numerical	Both	Photosynthetic pigment
Photosynthetic efficiency (Fv/Fm)	Photosynthesis	Dimensionless	Numerical	Both	Measures the efficiency of photosystem II
Chl *a/c*2 ratio and chl‐*a*/carotene	Photoprotection	dimensionless	Numerical	Both	Indicates adaptation to different irradiance conditions
Mycosporine‐like amino acids content	Photoprotection	nmol mg^−1^ protein	Numerical	Both	Indicates adaptation to higher irradiance
Cell size	Cellular growth	Longest axis (μm)	Numerical	Both	Correlates with metabolic rate
Maximum specific growth rate^a^	Population growth	μ d^−1^	Numerical	In vitro	Measures ecological symbiont proliferation
Organic carbon storage capacity	Energy reserve and composition	μg cell^−1^	Numerical	Both	Indicates energetic potential
PUFA content	Energy reserve and composition	pg cell^−1^	Numerical	Both	Associated with membrane stability and stress tolerance
Elemental composition	Energy reserve and composition	Dimensionless	Numerical	Both	Indicates trade‐offs between nutrient availability and energy allocation
Carbon translocation	Symbiosis	μg mg^−1^ host protein	Numerical	In hospite	Quantifies symbiotic contribution to the host
Host specificity	Symbiosis	Number of host species	Categorical	In hospite	Indicates degree of specialization
Lifestyle (free‐living/symbiotic)	Symbiosis	n/a	Categorical	Both	Defines the ecological mode of interaction
Nitrogen acquisition and adaptation to changing concentrations	Nitrogen assimilation	μmol g^−1^ h^−1^ (*V*_max_), μmol L^−1^ (*K*_*m*_), mol L^−1^ (*C*_min_)	Numerical	Both	Metabolism and cellular synthesis
Bathymetric amplitude	Ecological plasticity	m (depth of occurrence interval)	Numerical	In hospite	Indicates tolerance to light variation across depth gradients
Turbidity adaptation	Ecological plasticity	m^−1^ (Kd490)	Categorical	Both	Associated with survival in marginal reef environments
Geographic range	Ecological plasticity	km^2^ (area of occurrence)	Numerical	In hospite	Measures spatial ecological success
Growth rate under thermal stress	Thermal tolerance	μ d^−1^	Numerical	In vitro	Evaluates thermal resilience
Heat‐induced photobleaching	Thermal tolerance	Symbiont cells lost mg^−1^ host protein or reduction in Fv/Fm	Numerical	Both	Evaluates thermal sensitivity within host
ROS production under thermal stress	Thermal tolerance	au (probe fluorescence) over time	Numerical	Both	Indicates risk of enzyme and photosystem damage

*Note:* The traits are organized according to their primary biological function, suggested measurement unit, data type (numerical or categorical—see Section 4), whether they can be experimentally assessed in hospite, in vitro, or both, and ecological relevance. *V*_max_ = maximum uptake rate, *K*_*m*_ = Michaelis–Menten constant, *C*_min_ = minimum concentration.

Abbreviations: Chl, chlorophyll; PUFA, polyunsaturated fatty acids; ROS, reactive oxygen species.

^a^ The specific growth rate (*μ*) quantifies the proportional increase in cell concentration per unit time during exponential growth, calculated as the slope of ln(cell concentration) versus time.

### 5.1 Photosynthesis

The main ecological process driven by Symbiodiniaceae is photosynthesis, which supports primary productivity in reef environments. We propose the adoption of two commonly measured functional traits related to the photosynthetic process: (i) chlorophyll‐*a* (chla) content, a photosynthetic pigment, which serves as a proxy for microalgae biomass and is also a key indicator of ecological disturbances (Kalaji et al. 2017; Xu et al. 2025); and (ii) the maximum quantum yield of Photosystem II (Fv/Fm), used to quantify the efficiency of light energy conversion during photosynthesis, which serves as a critical diagnostic measurement of symbiont health and can be readily measured with a portable fluorometer (Kangasjärvi et al. 2014; Amario et al. 2023).

### 5.2 Photoprotection

Photoprotection refers to any process that reduces damage from ultraviolet radiation (which is key in shallow and well‐lit reef environments). We propose two photoprotection traits: (i) chlorophyll *a/*c2 and chlorophyll/carotens ratios, which indicate the relative abundance of light‐harvesting pigments and are associated with photoacclimation strategies, since these pigments absorb light in a different form (Oakley et al. 2022; Martins et al. 2023); and (ii) mycosporine‐like amino acid concentrations, which are photoprotective compounds that absorb ultraviolet radiation and mitigate oxidative stress through reactive oxygen species (ROS) scavenging, thereby reducing cellular damage and enhancing symbiont stress tolerance under high irradiance conditions (Banaszak et al. 2006; Rosic and Dove 2011; Rosic 2012).

### 5.3 Cellular Growth

We propose one functional trait pertaining to physiological growth: (i) individual cell size (diameter or longest axis), which influences metabolic processes and nutrient uptake in the symbiont and can enable flexible mechanisms in a changing environment (Kemp et al. 2020; Fung and Bergmann 2023). From individual cell size, other relevant metrics may be derived, such as volume.

### 5.4 Population Growth

We propose one functional trait for population growth: (i) maximum specific growth rate under controlled conditions, estimated in vitro across an ecologically relevant thermal range. This trait reflects the intrinsic proliferative capacity of a given Symbiodiniaceae lineage and serves as a comparative proxy for colonization potential and ecological performance (Klueter et al. 2017; Cui et al. 2022). However, specific growth rate is limited to lineages that can be cultured in vitro (in culture conditions) and does not account for in hospite (within the host) dynamics.

### 5.5 Energy Reserves and Composition

Energy reserves in Symbiodiniaceae can buffer short‐term fluctuations in photosynthesis and serve as a major source of translocated carbon to the coral host, directly influencing coral growth, reproduction, and calcification (Wang et al. 2015; Cunning et al. 2017). We propose three traits for this function: (i) organic carbon storage capacity, essential for maintaining energy balance under stress and closely linked to symbiont resilience and carbon retention capacity (Ros et al. 2020); (ii) polyunsaturated fatty acids (PUFA) content, which support membrane fluidity, thermal stress, and oxidative stress tolerance, functioning as vital components of energy storage, growth, reproduction, and symbiotic signaling (Imbs et al. 2014; Botana et al. 2022); and (iii) elemental composition (i.e., ecological stoichiometry, addressed through “elementome” assessments—see Camp et al. 2025), which characterizes the balance of cellular elemental ratios including key macronutrients such as carbon, nitrogen, phosphorus, and calcium as well as a suite of micronutrients and trace metals (e.g., iron, manganese, strontium, zinc, and copper). This serves as an integrative measure of nutrient acquisition, metabolic allocation, as well as trade‐offs between environmental nutrient availability and energy storage (Meunier et al. 2017; Luza et al. 2023).

### 5.6 Symbiotic Relationship

In a functional photosymbiosis, both partners regulate nutrient and carbon exchange in ways that promote mutual benefit and ensure stability. We propose three relevant traits: (i) carbon translocation (in hospite), to quantify the amount of photosynthates transferred from the symbiont to the host (Tremblay et al. 2014; Ros et al. 2021; Ianniello et al. 2025); (ii) host specificity, a species trait, which indicates the degree of specialization, which is important because generalist symbiont lineages may offer resilience by colonizing multiple hosts, while specialists often show tighter metabolic integration with their limited hosts (Berkelmans and Van Oppen 2006; Thornhill et al. 2009); this trait can be defined by the number of host species a symbiont lineage associates with; and (iii) predominant lifestyle (symbiotic or free‐living), to address whether a given Symbiodiniaceae lineage has been recorded as the dominant mutualistic partner within a host or seems to exist primarily in a free‐living state (Mohamed et al. 2020; Fujise et al. 2021; Li et al. 2024).

### 5.7 Nitrogen Assimilation

Nitrogen assimilation is a key regulator of Symbiodiniaceae growth and metabolism because it is required for the synthesis of proteins, nucleic acids, and photosynthetic machinery (Davy et al. 2012; Kopp et al. 2013; Xiang et al. 2020). In coral–algal symbioses, nitrogen availability strongly influences carbon allocation (Baker et al. 2013; Wong et al. 2021; McIlroy et al. 2022), with limitation often promoting carbon translocation to the host (Morris et al. 2019; Rädecker et al. 2021). Conversely, nitrogen enrichment can destabilize this balance by increasing symbiont proliferation and reducing host energy supply (Rädecker et al. 2021). Nitrogen status also modulates photosynthetic performance and stress tolerance, linking nutrient dynamics directly to bleaching susceptibility (Baker et al. 2013; Nitschke et al. 2022). As a result, nitrogen assimilation functions as a central control point in the regulation and stability of the coral holobiont. We propose one functional trait within the nitrogen assimilation function, which is (i) nitrogen acquisition and adaptation to changing concentrations (recorded as *V*_max_, *K*_*m*_, and/or *C*_min_—only measurable in vitro), which measures the ability of Symbiodiniaceae to modulate physiology in response to changing nitrogen levels, influencing growth and photosynthesis (Lesser 1996; Morris et al. 2019).

### 5.8 Ecological Plasticity

We propose the following four traits, which relate to how Symbiodiniaceae respond to environmental conditions. They are (i) bathymetric amplitude (the depth range over which the lineage has been observed), which reflects exposure to different light regimes, indicating tolerance to light variation across depth gradients (Bongaerts et al. 2015; Camp et al. 2020); (ii) turbidity adaptation, which allows for Symbiodiniaceae survival in marginal reef environments by enabling efficient photosynthesis under low‐light conditions, thereby also supporting coral persistence where light is limited (Millán‐Márquez et al. 2024; Toullec et al. 2024); lineages may be considered as clear‐water specialists if found in areas with Kd490 (a proxy for turbidity and underwater light attenuation) value of ≤ 0.1 m^−1^, while turbidity‐adapted species occur under higher Kd490 conditions (Shi and Wang 2010); and (iii) geographic range, an indicator of ecological success across widespread environments (Pontasch et al. 2014; Sturm et al. 2022).

### 5.9 Thermal Tolerance

We propose three traits related to thermal tolerance given the importance of this functional trait in the context of the climate crisis: (i) maximum specific growth rate under thermal stress (in vitro), because growth performance at elevated temperatures serves as a good proxy for physiological tolerance to heat (Chakravarti and van Oppen 2018; Zweifler et al. 2024); (ii) heat‐induced photobleaching (in hospite), which reflects the susceptibility of Symbiodiniaceae to thermal stress, where lineages that preserve photosystem function can mitigate bleaching (Maruyama et al. 2022; Wang et al. 2023); and (iii) production of ROS (in vitro), a direct measure of oxidative stress in the algal cell (Amario et al. 2023). Standardized assessments are especially important in the case for these traits (Grottoli et al. 2021). While exposure to temperatures 3°C above the local maximum monthly mean may be ecologically relevant under projected climate change scenarios (IPCC 2021), the use of thermal performance curves would provide a more comprehensive characterization of thermal tolerance (Dilernia et al. 2023; Scharfenstein et al. 2024). Alternatively, thermal stress could be standardized using anomaly accumulation metrics such as Experimental Degree Heating Weeks (eDHW), or shorter‐duration variants including Degree Heating Days (DHD) and Degree Heating Hours (DHH) (Leggat et al. 2022; Voolstra et al. 2025), thereby facilitating comparisons across studies and species.

The proposed set of 19 functional traits offers an integrative framework for beginning to understand the ecological roles and adaptive capacities of Symbiodiniaceae. These traits span a wide range of biological functions, from core metabolic processes to ecological plasticity, and are selected for their measurability and direct relevance to symbiont performance under environmental stressors. Together, these traits form a coherent and operationally measurable suite for trait‐based ecology in Symbiodiniaceae, supporting the development of more accurate and predictive models of coral‐symbiont dynamics under global environmental change. Rather than representing isolated functions, the nine trait categories should be viewed as interacting components of an integrated physiological network that collectively determines Symbiodiniaceae performance and ecological success.

Importantly, these 19 traits have already been measured for several Symbiodiniaceae lineages and corresponding data is available. Using the *Symbiodinium microadriaticum* species as a study case, multiple of these traits have been assessed: chlorophyll‐a (~3.0 pg chl‐a cell^−1^—Hennige et al. 2009), photosynthetic efficiency (Fv/Fm of 0.5—Oakley et al. 2023), cell size (up to ~13 μm—Lee et al. 2015), maximum specific growth rate (0.34 μm day^−1^—McLenon and DiTullio 2012), host specificity (it is a generalist species—Mies et al. 2020), lifestyle (it can be found both free‐living and in symbiosis—LaJeunesse 2001; Takabayashi et al. 2012), bathymetric amplitude (0–20 m—Ziegler et al. 2015), carbon translocation (78% of photosynthetically fixed carbon was transferred to host *Stylophora pistillata* after 48 h—Tremblay et al. 2012), growth rate under thermal stress (−2.2 to 1.7 μm day^−1^ under a ramp up to +8°C—Arossa et al. 2026), and ROS production under thermal stress (12.5 fluorescence units of probe per cell volume for overall ROS at +9°C for 4 h—Wietheger et al. 2018).

It is important to emphasize that functional traits in Symbiodiniaceae are inherently context‐dependent. The expression and ecological relevance of a given trait may vary across environmental gradients, host identity, life‐history stage, and whether the organism is studied in hospite or in vitro (Suggett et al. 2017; Rädecker et al. 2021; Oakley and Davy 2018; Aichelman et al. 2024). Although presented individually for clarity and standardization, many traits are physiologically interconnected and should not be viewed as independent dimensions of performance. For example, photosynthetic efficiency, pigment composition, photoprotective compounds, and reactive oxygen species production are linked through mechanisms regulating light harvesting and photodamage. Such trait covariance is common in functional ecology (Walker et al. 2017) and can be useful as it often reveals broader functional patterns. Future studies may therefore benefit from multivariate approaches that identify major axes of functional variation and trait relationships.

The next step for guiding future trait‐based research is to compile global‐scale data on these 19 traits for Symbiodiniaceae lineages and integrate them into a unified database. Given the context dependence, a future trait database should systematically include ecological and experimental metadata (e.g., life stage, host association, and culture conditions) to ensure correct interpretation and comparability of trait values, as well as taxonomic identifiers where available.

## 6 Challenges for Assessing Symbiodiniaceae Functional Traits

Developing functional frameworks is particularly challenging due to the unique biological characteristics of Symbiodiniaceae, particularly while engaged in endosymbiosis. Unlike corals or fishes, these dinoflagellates pose inherent difficulties to conventional trait‐based approaches, which are typically tailored to macroscopic organisms with readily observable morphological or behavioral traits. Symbiodiniaceae exhibit limited morphological variability, especially when inside the host, with most lineages appearing identical under optical microscopy, thereby limiting the utility of cell morphology for ecological inference (Suggett et al. 2017; LaJeunesse et al. 2018). Instead, their functional identity and ecological roles are primarily defined by physiological and biochemical attributes, including thermal tolerance, nutrient uptake, and oxidative stress responses. For example, distinct lineages may vary markedly in their production of ROS, growth rates, pigment profiles, lipid and metabolite composition, as well as mortality under environmental stressors, such as heat or light exposure (Tchernov et al. 2004; Parkinson and Baums 2014; Goyen et al. 2017; Botana et al. 2022; Amario et al. 2023). These traits, while functionally meaningful, often require specialized analytical methods for measurement.

Assessing Symbiodiniaceae functional traits can be approached in hospite, ex hospite (free‐living in the environment), or in vitro, with each method offering distinct strengths and limitations. In vitro assays provide greater experimental control and enable the isolation of symbiont‐specific responses without host interference, thereby enabling precise measurements of photosynthetic performance, ROS production, and cell division under controlled conditions (Goyen et al. 2017). However, such artificial settings do not accurately capture the complex physiological interactions between host and symbiont that influence holobiont performance in situ. Furthermore, it is important to keep in mind that these in vitro responses are related to the culture conditions (type of medium, pH, and irradiance) and by the growth phase assessed. Moreover, nutrient‐replete media are not representative of the nutrient‐limited conditions most Symbiodiniaceae experience within host cells (Maruyama and Weis 2021; Herrera et al. 2021). Therefore, in hospite assessments may provide a more ecologically relevant context, preserving the metabolic coupling, signaling, and feedback mechanisms that occur within the holobiont (Fitt et al. 2001; Tchernov et al. 2004). Yet, this ecological realism comes with trade‐offs, where host‐mediated effects on nutrient availability, immune modulation, and cellular homeostasis can obscure the attribution of specific traits to the symbiont alone (Weis 2008; Davy et al. 2012; Parkinson et al. 2016; Aichelman et al. 2024).

Another key challenge lies in the isolation and maintenance of Symbiodiniaceae strains under laboratory conditions, which is essential for in vitro experimentation, but often proves difficult due to the specific physicochemical requirements of different symbiodiniaceans (Krueger and Gates 2012; Fujise et al. 2018; Rodriguez and Ho 2018; Romero et al. 2022). In hospite measurements present additional challenges, as they demand careful sampling techniques that preserve the integrity of the host–symbiont association. These technical barriers are compounded by persistent gaps in the understanding of Symbiodiniaceae diversity. Although recent phylogenomic efforts have uncovered extensive diversity, the functional implications of this molecular variation remain largely uncharacterized (LaJeunesse et al. 2018; Quigley et al. 2019; Dougan et al. 2022). As a result, field‐based trait research on Symbiodiniaceae remains relatively scarce compared to that of reef fishes and scleractinian corals, which benefit from more accessible morphofunctional frameworks and a longer history of ecological trait assessment (Mouillot et al. 2014; McWilliam et al. 2018).

An additional challenge is the substantial phenotypic variation that may occur among strains, genotypes, or ITS2 lineages belonging to the same Symbiodiniaceae species. Traits can vary considerably among closely related isolates, sometimes approaching or exceeding differences observed among species (Hawkins et al. 2016; Díaz‐Almeyda et al. 2017; Hoadley et al. 2021). This variability is most readily quantified in vitro, where clonal cultures can be maintained under controlled conditions and host effects minimized. Rather than treating such variation as a source of noise, future trait databases should preserve measurements at the finest possible taxonomic resolution and record associated environmental and methodological metadata. Trait values can then be aggregated hierarchically across strains, species, and genera depending on the ecological question and level of taxonomic confidence. Such an approach would allow the framework to explicitly incorporate intraspecific variation while maintaining compatibility with broader functional analyses. Within this context, an important intermediate step toward the development of a comprehensive Symbiodiniaceae functional trait database will be the systematic synthesis of existing trait information scattered throughout the scientific literature. Such efforts will also need to evaluate the extent of taxonomic coverage, compare measurements obtained in hospite and in vitro, identify methodological inconsistencies among studies, and quantify the availability of data across different functional categories. However, the reliability of any large‐scale synthesis will depend on robust taxonomic curation, as historical inconsistencies in lineage designation complicate the attribution of trait values to specific Symbiodiniaceae taxa.

To ensure meaningful comparisons across studies and lineages, functional trait assessments in Symbiodiniaceae must be conducted using standardized methods and clearly defined protocols. Each trait should ideally be linked to a specific, reproducible measurement, obtained with calibrated instruments that quantify physiological performance under controlled conditions (Suggett et al. 2015; Oakley and Davy 2018). Furthermore, trait measurements should be performed under environmental conditions that closely resemble those experienced by the organism in its natural habitat, particularly regarding light and temperature, to minimize confounding stress effects and restrict the phenotypic range to the inherent functional potential of the organism rather than stress responses (Warner et al. 1996; Goyen et al. 2017). However, when assessing traits related to stressors, experimental conditions must replicate the stressor itself (and potentially interactions with other stressors) to elicit meaningful functional responses. Recent technological advances now offer new avenues to quantify functional diversity more precisely, especially through high‐resolution molecular tools. Genomic, transcriptomic, proteomic, metabolomic, and lipidomic platforms enable detailed characterization of symbiont function and plasticity across environmental gradients, often revealing cryptic but ecologically significant trait variation (Aranda et al. 2016; González‐Pech et al. 2021; Botana et al. 2022, 2025; Roach et al. 2025; Timmins‐Schiffman et al. 2025). Overcoming these challenges in Symbiodiniaceae functional ecology will ultimately require the creation of a centralized, accessible, and ecologically meaningful trait database. Ideally, a comprehensive trait repository would include parallel in hospite and in vitro trait records for each lineage where possible.

## 7 Perspective and Future Directions

**FIGURE 4:**
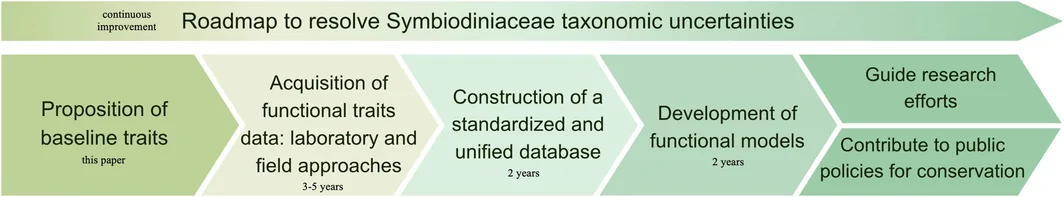
Conceptual roadmap for advancing trait‐based research in Symbiodiniaceae. The framework illustrates a sequential process that begins with the proposition of ecologically relevant baseline traits (see Section 4), followed by the acquisition of functional data under both laboratory and field conditions. These datasets should then be compiled into a standardized and unified database that integrates taxonomic, physiological, and ecological information across lineages. The final step involves the development of functional models capable of quantifying functional diversity and redundancy. In parallel with continuous progress in resolving taxonomic uncertainties, this roadmap provides a foundation for identifying vulnerable or functionally irreplaceable assemblages, thereby improving early prediction of ecosystem decline, refining conservation prioritization, and informing decision‐makers and stakeholders. The estimated time to complete each step is provided.

Advancing Symbiodiniaceae research will require a coordinated framework that integrates foundational taxonomy with trait‐based ecological approaches (Figure 4). A first critical and challenging step is to resolve the persistent taxonomic uncertainty surrounding much of Symbiodiniaceae diversity, as numerous lineages remain undescribed or unassigned to formal species, thereby constraining efforts to associate ecological functions with specific symbiont identities (Davies et al. 2023). A refined and comprehensive taxonomic baseline, including the formal description of new species and the systematic classification of lineages, is essential. In parallel, fundamental research must prioritize the generation of trait data for both ecologically abundant and rare Symbiodiniaceae lineages. Such effort requires both laboratory‐based experimentation and in situ fieldwork to capture trait expression across natural and controlled environments. Data generated from these efforts should contribute to the creation of a centralized, open‐access functional trait database for Symbiodiniaceae, designed after established platforms for reef corals and fishes. Moreover, the expansion of trait‐based modeling across broader spatial scales will enable the quantification of key ecological parameters, such as functional diversity, redundancy, and vulnerability. This will facilitate systematic comparisons across reef regions and environmental gradients. Ultimately, such a framework will enhance decision‐making in coral reef conservation by offering insights into ecosystem vulnerability or resilience, anchored not only in coral responses but also in those of their primary symbiotic partners. As biological indicators of thermal stress tolerance, Symbiodiniaceae provide a critical yet underutilized perspective for forecasting the stability and future of coral reef ecosystems. Given their central role in coral health and their response to temperature fluctuations, it is fundamental that future models incorporate functional approaches to Symbiodiniaceae to better understand and predict reef resilience under climate change.

## Author Contributions

**Luna Mayura F. Bauer:** conceptualization (equal), writing – original draft (equal). **Michelle Amario:** writing – original draft (equal). **Luiza P. Campos:** writing – original draft (equal). **Aline C. Shimada:** writing – original draft (equal). **Marina T. Botana:** writing – original draft (equal). **Sarah W. Davies:** writing – original draft (equal). **Amana G. Garrido:** writing – original draft (equal). **Arthur Z. Güth:** writing – original draft (equal). **Guilherme O. Longo:** writing – original draft (equal). **André L. Luza:** writing – original draft (equal). **Matthew R. Nitschke:** writing – original draft (equal). **John E. Parkinson:** writing – original draft (equal). **Flávia M. P. Saldanha‐Corrêa:** writing – original draft (equal). **Christian R. Voolstra:** writing – original draft (equal). **Carla Zilberberg:** writing – original draft (equal). **Miguel Mies:** conceptualization (equal), writing – original draft (equal).

## Conflicts of Interest

The authors declare no conflicts of interest.

## Data Availability

The authors have nothing to report.
